# Evaluation of Risk Factors for Periprosthetic Joint Infection Following Reverse Shoulder Arthroplasty: A Multivariate Analysis Study

**DOI:** 10.3390/jcm14092926

**Published:** 2025-04-24

**Authors:** Koray Şahin, Hakan Batuhan Kaya, Cemil Burak Demirkıran, Nezih Ziroğlu, Christos Koukos, Vahdet Uçan, Mehmet Kapıcıoğlu, Kerem Bilsel

**Affiliations:** 1 Department of Orthopedics and Traumatology, Bezmialem Vakif University, Istanbul 34093, Turkey; parisabatu@gmail.com (H.B.K.); vahdetucan@hotmail.com (V.U.); kapicioglum@gmail.com (M.K.); 2 Department of Orthopedics and Traumatology, Saray State Hospital, Tekirdağ 59600, Turkey; drburakdemirkiran@gmail.com; 3 Department of Orthopedics and Traumatology, Acibadem University Atakent Hospital, Istanbul 34303, Turkey; nezih.ziroglu@gmail.com; 4 Sports Trauma and Pain Institute, 54655 Thessaloniki, Greece; koukos_christos@hotmail.com; 5 Department of Orthopedics and Traumatology, Acibadem University Fulya Hospital, Istanbul 34349, Turkey; kbilsel@gmail.com

**Keywords:** shoulder joint, infection, arthroplasty, prosthesis-related infections, diabetes mellitus, C-reactive protein

## Abstract

**Background/Objectives**: Reverse shoulder arthroplasty (RSA) has been widely used for the treatment of shoulder pathologies, particularly rotator cuff tear arthropathy. Currently, it is also increasingly performed for different indications. Like in any arthroplasty procedure, periprosthetic joint infection (PJI) is one of the most concerning complications and may have devastating outcomes. This study aimed to identify risk factors for PJI following RSA. **Methods**: This retrospective case-control study was conducted with patients who underwent RSA during the study period. Based on PJI occurrence during the follow-up period, patients were divided into two groups: Group I (no infection) and Group II (infection). The relationship between numerous clinical variables and PJI was tested. All variables were initially evaluated through univariate analysis between the two groups, and variables showing significant differences between the two study groups were subjected to multivariate logistic regression analysis to determine independent risk factors. **Results**: The study included 302 patients, with a mean age of 69.6 ± 10.1 years and a mean follow-up duration of 59.8 ± 24.7 months. During the follow-up period, PJI was not detected in 289 patients (95.7%) (Group I), while 13 patients (4.3%) developed PJI (Group II). Univariate analysis revealed a significant association between preoperative C-reactive protein (CRP) value (*p* = 0.001) and preoperative diabetes history (*p* = 0.007) with PJI. Multivariate logistic regression analysis, including these two variables, showed that diabetes was an independent risk factor for PJI development (*p* = 0.01, odds ratio = 4.85). Preoperative CRP elevation was not observed as an independent risk factor. **Conclusions**: This study demonstrated a significant association between high preoperative CRP levels and diabetes with PJI. Additionally, the presence of diabetes was identified as an independent risk factor for infection, with a 4.85-fold higher risk of PJI development in patients with a history of diabetes.

## 1. Introduction

Since its first introduction for the treatment of cuff tear arthropathy (CTA), reverse shoulder arthroplasty (RSA) has shown successful and reliable outcomes [1]. In the following decades, indications for RSA have expanded, including proximal humeral fractures, fracture sequelae, glenohumeral osteoarthritis (OA), failed prior rotator cuff repair, avascular necrosis (AVN) of the humeral head, revision of failed prior shoulder arthroplasty etc. [2] Even though clinical outcomes have been promising for these various pathological conditions [1,3]; high complication rates have been attributed with RSA including periprosthetic joint infection (PJI), instability, fracture, scapular notching, aseptic loosening or neurological injury [4].

As in all arthroplasty procedures, PJI is a major concern following RSA, which may require multiple additional surgeries and long-duration antibiotic treatment. In previous reports, PJI incidence following primary RSA ranges from 0.5 to 6.7%, but most commonly between 3 to 4% [5,6,7]. The current knowledge regarding PJIs is mainly based on studies about lower limb arthroplasty. The proper definition of PJI, identification of risk factors, and a precise diagnosis can ensure timely care with favorable outcomes, but they continue to pose a significant challenge. Recent proposals for stepwise diagnosis algorithms incorporate a combination of medical features, serum and synovial tests, along with histological and microbiological evaluations [8]. The definitions of PJI have undergone multiple revisions over the years, with the most recent and significant ones articulated by the International Consensus Meeting (ICM) on Musculoskeletal Infections in 2018 [9] and the European Bone and Joint Infection Society (EBJIS) in 2021 [10]. Earlier definitions of PJI characterized the outcomes in a “binary” manner, indicating whether an infection was present or absent. In current definition systems, the outcomes can be presented across a broader range of probabilities, including categories like “definite/probable/possible/unlikely infection” or “confirmed/likely/unlikely infection”. This offers a significant benefit in patient assessment.

Serum biomarkers like C-reactive protein (CRP), erythrocyte sedimentation rate (ESR), white blood cell (WBC) count, and α-defensin have been incorporated as parameters for PJI detection in these current diagnostic criteria [9,10] since they can be obtained non-invasively and due to their importance as indicators of the inflammatory process in PJI. Nonetheless, their effectiveness is still open to investigation. A recent study [11], which was conducted with hip arthroplasties, investigated the diagnostic value of numerous inflammatory biomarkers to diagnose and differentiate PJI from aseptic failure. Their results showed that systemic inflammation index (SII) and neutrophil/lymphocyte ratio (NLR) were the most precise biomarkers for PJI detection. Additionally, several other serum biomarkers were identified as being independently associated with PJI diagnosis. Numerous prior investigations have investigated various biomarkers, including serum procalcitonin, interleukin-6 (IL-6), tumor necrosis factor-α (TNF-α), D-dimer, serum platelet count, serum fibrinogen levels and many others to assess their diagnostic significance in PJI detection [12,13,14]. Nonetheless, the outcomes of these investigations vary significantly. Furthermore, as previously noted, the majority of these studies concentrate on lower extremity arthroplasty, while the understanding of shoulder arthroplasty remains significantly restricted. Consequently, many possibilities for future investigation remain regarding PJI that develops following shoulder arthroplasty. Due to the increasing demand for RSA and growing concerns for subsequent PJIs, the determination of risk factors related to PJIs is essential. However, there is a paucity of evidence in the literature regarding patient and procedure-related risk factors on PJIs following RSA, and most of the available data is confined to certain factors without controlling for confounding variables [3,15,16,17]. This brings out the need for further investigation with a large series of patients and with multiple variable analyses in order to confirm independent risk factors for PJIs.

This study aims to investigate the association of patient and procedure-related factors with PJI development and to identify independent risk factors for PJI following RSA.

## 2. Materials and Methods

This study was a retrospective analysis of prospectively collected data from patients who underwent RSA surgery between April 2014 and May 2022. It was conducted in a tertiary university hospital clinic. All patients included were informed about the use of their medical records data for publication, and written informed consent was obtained.

### 2.1. Inclusion and Exclusion Criteria

Records of patients who underwent RSA surgery during the study period were assessed from a prospectively collected institutional shoulder arthroplasty database. All interventions were performed by a single surgeon, who is a fellowship-trained, experienced shoulder surgeon. Patients who had a history of any kind of infection (septic arthritis, osteomyelitis) in the operative shoulder, patients who underwent RSA procedure as a revision of a prior failed shoulder arthroplasty (RSA, total anatomic shoulder arthroplasty or hemiarthroplasty), patients with a history of any rheumatoid condition or immunodeficiency and patients who received immunosuppressive treatment during the follow-up period were excluded. Included patients were sub-grouped per diagnosis for RSA indication in order to assess the influence of diagnosis on PJI, and five different diagnoses were identified: CTA, proximal humeral fracture (PHF), glenohumeral OA, sequelae of previously operated PHFs (malunion or nonunion), AVN of the humeral head and failed prior arthroscopic rotator cuff repair (fARCR).

### 2.2. Surgical Technique

All patients were operated on under general anesthesia with preoperative interscalene nerve block on the beach-chair position. The skin preparation was performed using a povidone-iodine paint solution combined with isopropyl alcohol. The operative field was covered with Ioban 2 surgical drapes (3M, St Paul, MN, USA), and no surgical helmets were used during the intervention.

A standard deltopectoral approach was preferred, and uncemented humeral stems were used for all interventions. Suction drains or medical prophylaxis for deep venous thrombosis were not routinely used. Deep venous thrombosis prophylaxis was performed using compressive stockings.

### 2.3. Postoperative Rehabilitation

Immobilization using an abduction sling with the shoulder in neutral rotation and 30 degrees of abduction was performed for all patients for 4 weeks. Passive and active elbow and wrist motions were encouraged immediately after surgery. In the 4th postoperative week, passive shoulder range of motion (ROM) exercises were initiated and continued until full passive ROM was achieved combined with active assisted ROM exercises. In the 6th to 8th postoperative week, active ROM, deltoid strengthening and shoulder proprioception exercises were initiated. Return to full physical activity was allowed considering the recovery level of each patient starting from the 3rd to 6th postoperative month.

### 2.4. Infection Prophylaxis Protocol

All patients were screened preoperatively for possible urinary tract infection (UTI) with urinalysis, and further urine cultures were obtained to see if the results of the urinalysis were consistent with the UTI. These patients were consulted by the infectious disease department for appropriate treatment, and surgeries were delayed considering their counseling.

On the operative day, 2 g of intravenous (IV) cefazolin was administered 30 min before incision for all patients, and an additional 4 doses of 1 g IV cefazolin were continued every 6 h of the first postoperative 24 h. In 2020, the antibiotic protocol was changed, and an additional local administration of 2 g of teicoplanin in powder format into the surgical area before wound closure was performed routinely for all patients.

### 2.5. Clinical Evaluation

All indications for RSA were given by the senior surgeon following preoperative clinical and radiological assessment. All demographic and clinical data of patients were prospectively collected in a shoulder arthroplasty database. Postoperative clinical evaluations were performed in the 2nd week, 4th week, 3 months, 6 months, and 12 months postoperatively and each following postoperative year. All intraoperative and postoperative complications were noted.

All patients with suspicious clinical findings for PJI during the follow-up period underwent an infection workup. The findings that cause an infection suspicion include new onset of pain or persistent postoperative pain without other explanation, a sinus tract around the surgical wound, persistent erythema and/or fluctuance around the surgical wound and persistent wound drainage. Criteria described by the International Consensus Meeting (ICM) for shoulder PJI [9] were used for diagnosis (Table 1). The presence of a major criterion (definite PJI) or probable PJI according to minor criteria was considered as PJI. Patients that were classified as possibly PJI were followed up until the resolution of the clinical findings or diagnosed as PJI if it was confirmed by intraoperative findings and intraoperatively obtained cultures.

Patients without a PJI diagnosis constituted group I, and patients with a PJI diagnosis constituted group II. Demographic and clinical variables were compared between two study groups with a univariate analysis in order to find out which variables were associated with PJIs. A multivariate analysis was then conducted with the significant variables of the univariate analysis in order to confirm which variables were independent risk factors for PJI.

### 2.6. Data Collection

The variables extracted from the department’s database for statistical analysis included age, gender, body mass index (BMI), duration of hospital stay, duration of surgical intervention, preoperative laboratory analysis (hemoglobin, hematocrit, CRP, ESR, glucose, hemoglobin A1c levels and urinalysis), indication for RSA surgery (CTA, PHF, glenohumeral OA, fracture sequelae, AVN, fARCR), intraoperative local teicoplanin application, tobacco consumption and associated comorbidities including diabetes mellitus, hypertension, chronic obstructive pulmonary disease (COPD) and coronary artery disease.

Analyzed preoperative glucose levels consisted of fasting blood glucose measurements obtained on the morning of the surgery. Hemoglobin A1c (HbA1c) data were not available for a majority of the study cohort. However, medical records of the patients showed that all patients who had prior diabetes mellitus diagnosis and who were under treatment for diabetes had been routinely screened for HbA1c levels, and these data were used for analysis.

### 2.7. Statistical Analysis

Mean, median, standard deviation, range, percentage and frequency were determined as descriptive statistical methods in order to analyze study data. The distribution of normality of continuous data was tested using the Shapiro–Wilk test, Kolmogorov–Smirnov test and histograms. The univariate analysis was conducted using an unpaired *t*-test for continuous variables and a chi-square or Fisher’s exact test for categorical variables. A comparison of non-normally distributed continuous data was performed using the Mann–Whitney U test.

A multivariate logistic regression analysis was then conducted with statistically significant variables observed in the univariate analysis in order to determine independent risk factors and to estimate odds ratios (ORs) for PJI. All analyses were performed using SPSS Statistics for Windows (Version 26.0.0, Armonk, NY, USA), and the significance level was set at *p* = 0.05.

## 3. Results

**Figure 1:**
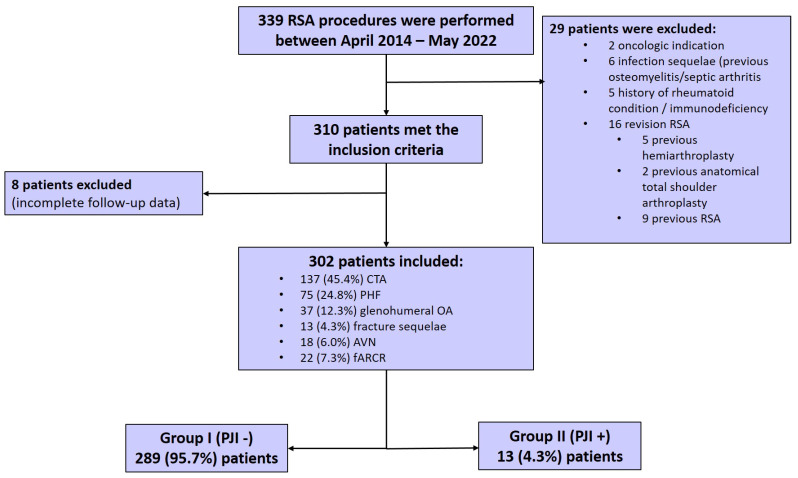
Patient selection flowchart diagram. Abbreviations: RSA: reverse shoulder arthroplasty, CTA: cuff tear arthropathy, PHF: proximal humeral fracture, OA: osteoarthritis, AVN: avascular necrosis, fARCR: failed arthroscopic rotator cuff repair, PJI: periprosthetic joint infection.

302 patients were included in the study, with a mean age of 69.9 ± 10.1 years and a mean follow-up duration of 59.8 ± 24.7 months. There were 232 (76.8%) female and 70 (23.2%) male patients. During the study period, 13 (4.3%) patients were diagnosed with PJI and constituted group II. No PJI was observed in 289 (95.7%) patients (group I). The distribution of patients according to preoperative diagnosis for RSA surgery was as follows: 137 (45.4%) patients with CTA, 75 (24.8%) patients with PHF, 37 (12.3%) patients with glenohumeral OA, 13 (4.3%) patients with fracture sequelae, 18 (6.0%) patients with AVN and 22 (7.3%) patients with fARCR (Figure 1).

Regardless of the limited number of PJI cases in our study (n = 13), a descriptive subgroup analysis was conducted based on the identified pathogens. The most frequently isolated microorganisms were *Cutibacterium acnes* and *Staphylococcus aureus* (both methicillin-sensitive and -resistant subtypes), each of which was identified in three patients. Subsequently, *Pseudomonas* spp. (n = 1), one polymicrobial infection (isolated organisms: *Morganella morgagnii* and Enterobacteriaceae) and two culture-negative infections were observed. In culture-negative infections, no pathogens could be identified despite apparent intraoperative purulence and preoperative laboratory findings suggesting PJI. Although no conclusive trends were established, *C. acnes* infections were observed to be clinically more subtle and identified in later stages during follow-up, indicating the indolent nature of this pathogen. On the contrary, *S. aureus* infection had more intense clinical manifestations. Structured statistical comparisons were unfeasible due to limited sample sizes. This descriptive analysis highlights the wide range of PJI pathogens in RSA and emphasizes the necessity for further studies on this topic.

A comparison of two study groups according to tested variables in the univariate analysis revealed that preoperative C-reactive protein (CRP) levels and history of diabetes were significantly associated with PJI (*p* < 0.05). The mean preoperative CRP level was 5.19 ± 13.9 mg/L in group I and 10.3 ± 10.4 mg/L in group II (*p* = 0.001). While there were 217 (75.1%) patients without diabetes history and 72 (24.9%) patients with diabetes history in group I, group II consisted of 5 (38.5%) patients without diabetes history and 8 (61.5%) patients with diabetes history (*p* = 0.007). No significant difference was observed between the two study groups for other tested variables (*p* > 0.05). Data of univariate analysis between the two study groups were resumed in (Table 2).

A subsequent multivariate logistic regression analysis was then performed with these two variables. The results of this analysis revealed that diabetes mellitus was the only independent risk factor for PJI, with a 4.85-fold increased risk (*p* = 0.007) (Table 3).

The glycemic control level of patients with diabetes diagnosis was assessed using preoperative HbA1c levels in this patient subgroup. There were 80 patients with a history of diabetes in total, of which 8 patients developed PJI. The comparison of mean preoperative HbA1c levels in diabetic patients in terms of PJI occurrence showed that HbA1c levels were significantly higher in patients with PJI (mean HbA1c levels were 7.45 ± 1.17 and 9.19 ± 0.68 respectively, *p* = 0.0001). This finding implies that the level of glycemic control might play a significant role in PJI development in diabetic patients. However, this variable could not be tested as a risk factor for PJI due to the unavailability of this data in non-diabetic patients.

## 4. Discussion

The findings of this study showed that high preoperative CRP levels and a history of diabetes were associated with PJI following RSA. Secondly, with an almost 5-fold increased risk, diabetes was found to be an independent risk factor for PJI.

In previous studies, PJI rates after RSA surgery were reported to be between 0.5 and 6.7%, but most commonly, they ranged from 3 to 4% [5,6,7]. In our series, the overall PJI rate was 4.3%, which was consistent with previous reports. The majority of current evidence suggests that there is an increased risk for PJI in males and in patients aged under 65 [5,18,19]. From a causative aspect, this is most likely multifactorial. However, it has been shown that the burden of *C. acnes* around the shoulder area was considerably higher in males, which might contribute to higher PJI rates in males [20]. Unlike this previous evidence, gender and age were not significantly associated with PJI in our series.

Diabetes is considered a major contributor to infection and impairment of wound healing following arthroplasty procedures; however, this knowledge relies mainly on reports about lower extremity arthroplasty. Current knowledge regarding the influence of diabetes on PJI following RSA surgery is rather limited and controversial. Moreover, most of the available data is confined to univariate analyses or retrospective database studies with low evidence levels. In a recent study, Rao et al. [21] reported a significant association between preoperative diabetes diagnosis and postoperative infection following shoulder arthroplasty. Additionally, they showed that while there was a significant association between elevated first postoperative glucose measurement and infection, second and third glucose level measurements were not found to be associated with infection. The authors stated that early postoperative glucose measurements represent the patient’s baseline glycemic control level more accurately, and they also concluded that strict postoperative glucose level control in diabetic patients might play a crucial role in infection prevention. Another retrospective database study reported that patients with an increased hemoglobin A1c level (>8 mg/dL) had a higher risk for wound complications and deep infection [22]. In contrast to these findings, there are numerous studies reporting no correlation between diabetes and PJI after shoulder arthroplasty [5,6,23,24]. However, the findings of the present study also suggest a significant association between preoperative diabetes history and PJI. Moreover, diabetes was found to be the sole independent risk factor for PJI (odds ratio: 4.85) among the tested variables. The findings of the present study indicated that preoperative serum glucose levels should not be considered a reliable indicator of glycemic control since we were unable to demonstrate a significant association between PJI occurrence and a single preoperative glucose level measurement. However, preoperative HbA1c measurements, which were available solely in diabetic patients, were significantly higher in patients who subsequently developed PJI. This finding highlights the importance of postoperative glycemic control. Nonetheless, further research with a larger series is mandatory to draw such a comprehensive conclusion.

Inflammatory markers such as CRP and erythrocyte sedimentation rate (ESR) are traditionally used for infection workup in clinical practice. These markers are not only considered primary diagnostic tools for PJI detection and included in diagnostic criteria described by ICM [9], but they are also used for preoperative screening for any underlying infectious condition before arthroplasty procedures. An increased preoperative level of these inflammatory markers is usually considered a relative contraindication for surgery and causes a delay in intervention. Current knowledge regarding accurate thresholds for CRP and ESR in order to enhance the sensitivity and specificity of these markers are based on previous reports studying hip or knee arthroplasties [25], and a significant paucity of evidence exist concerning the role and efficacy of inflammatory markers in shoulder PJIs. Low-virulent, indolent pathogens such as *C. acnes* constitute a considerable portion (38.9%) [26] of shoulder PJIs, and these infections might be a possible reason for the reduced sensitivity and specificity of these markers for shoulder PJI detection. An increased CRP or ESR level may also be due to other scenarios, such as reduced clearance of markers, an occult autoimmune condition or another infectious condition (UTI, upper respiratory system infection, etc.). Besides, patient-related factors, including female gender and older age, which constitute a large portion of the population who underwent RSA surgery, were reported to be associated with increased ESR levels in the healthy general population [27]. Considering all of these conditions, the use of preoperative inflammatory markers and their influence on risk assessment of shoulder PJI needs to be precisely clarified. A previous study by Kopechek et al. [28] examined risk factors for elevated preoperative CRP and ESR levels prior to shoulder arthroplasty surgery. Their findings showed that a considerable amount of patients undergoing shoulder arthroplasty surgery with a diagnosis of primary glenohumeral OA had elevated preoperative CRP (25.5% of patients) and ESR (29.8% of patients) levels. Unsurprisingly, they found out that active PJIs and acute PHFs caused a significant increase in preoperative CRP and ESR values compared to patients who underwent shoulder arthroplasty due to glenohumeral OA. The authors concluded that increased inflammatory marker levels were possible even without the presence of underlying infection, and elevated preoperative ESR and CRP levels might not be considered a relative contraindication for shoulder arthroplasty surgery. However, we think that current data is limited to support this implication, and more evidence is needed to draw such a conclusion. In contrast to these findings, our results showed that even though an increased preoperative CRP level was not an independent risk factor for PJI development, a significant association was present between preoperative CRP levels and PJI. These findings imply that surgeons should still be cautious about PJI development, and meticulous risk assessment with patient counseling is needed in case of an increased preoperative CRP level.

Future research on infection risk stratification in RSA should investigate the role of advanced biomarkers, including IL-6 and α-defensin. These indicators exhibit notable diagnostic efficacy in PJIs of the hip and knee, with IL-6 indicating early systemic inflammatory responses and α-defensin acting as a locally effective antimicrobial peptide, which is increased in the infected synovial fluid [11,13,29]. Although their function in shoulder arthroplasty is still being investigated, initial evidence indicates possible usefulness, especially in inconclusive situations [30,31,32]. Moreover, biofilm-associated infections, especially those induced by indolent pathogens like *Cutibacterium acnes*, present a considerable diagnostic challenge. These infections frequently evade identification by conventional culture techniques and may manifest with negligible symptoms [33]. Biofilm presence complicates diagnosis, diminishes antibiotic efficiency, and may lead to delayed or chronic infections. Subsequent research should focus on combining molecular diagnostics with biofilm-targeted assays to improve early diagnosis, especially in individuals with elevated inflammatory markers but inconclusive clinical findings or culture results.

According to available knowledge, revision arthroplasty surgery due to a failed prior shoulder arthroplasty is a known risk factor for PJI, with reported infection rates reaching up to 15.4% [15]. In the current study, revision RSA procedures were excluded, and we tried to focus on evaluating the influence of other diagnoses for RSA indication on PJI development. In previous reports, a history of previous non-arthroplasty shoulder surgery [16,34] and RSA surgery for PHF or its sequelae have been shown to have a higher risk for PJI [5,7,35]. However, there are also some reports suggesting that arthroplasty for trauma did not have any association or increased risk of infection [17,36]. In our series, we did not observe any influence of different indications on PJI. However, we still think that PJI might be more likely, especially when there is a history of previous shoulder surgery (prior rotator cuff repair or osteosynthesis of a PHF). Our study probably was unable to reveal these factors due to a low number of events. Therefore, future studies with larger cohorts are needed to clarify this issue.

Recently, interest in the intraoperative admission of antibiotics into the surgical wound for infection prevention has been raised. However, most of the available data on this issue is based on reports studying lower extremity arthroplasty or spine surgery, and evidence is quite limited for shoulder arthroplasty. A recent study by Iorio et al. [37] reported effects of a “vanco-povidone” protocol for patients with a high risk for infection following hip or knee arthroplasty. The protocol included lavage of the joint with a povidone-iodine solution after implantation, followed by 2 g vancomycin administration in powder form into the surgical wound before wound closure. They reported a 27.8% decrease in PJI rates in patients who received this protocol. In the 2018 ICM, it was suggested that intraoperative vancomycin powder administration might possibly have a role in infection prevention, but there was no available data on its use in shoulder arthroplasty. Subsequently, it was recommended with limited evidence in high-risk patients for PJI [38]. In our study, intraoperative teicoplanin powder was evaluated, and we could not reveal any significant positive effect on PJI prevention; therefore, we were unable to give any recommendation in favor of its use.

The primary drawback of the present study is the limited sample size in the PJI group (n = 13). The low occurrence rate, while aligned with the documented incidence of PJI in RSA, unavoidably restricts the statistical power of the analysis. Although the total cohort size (n = 302) is substantial, the disparity across groups (289 vs. 13) presents a risk of Type II error and diminishes the precision of impact estimates. This concern is especially pertinent to multivariate logistic regression, where a widely recognized guideline suggests a minimum of 10 events per variable (EPV) to secure model stability. In our model, we maintained an EPV value of 6.5 with two variables: diagnosis of diabetes and preoperative CRP level. The recent statistical literature indicates that satisfactory outcomes can be achieved with EPVs ranging from 5 to 9, contingent upon a limited number of predictors and meticulous model specification [39]. However, we recognize that a greater quantity of PJIs, preferably 30 or more, would yield more dependable results and facilitate the incorporation of further potentially pertinent factors. We chose to maintain the traditional significance level of *p* = 0.05, consistent with standard clinical research practices and other reports on this subject. Nevertheless, the findings, especially those derived from multivariate analysis, must be approached with caution and regarded as exploratory rather than conclusive. Additional multicenter studies with larger cohorts and higher PJI event rates are necessary to corroborate our findings and improve statistical reliability. The retrospective nature of this study is the second limitation that needs to be mentioned due to the possibility of selection bias or other unanticipated factors that may have distorted the results. However, prospectively collected information from an arthroplasty database was used, which could minimize this risk. Another limitation is that the data on preoperative HbA1c levels was restricted to a specific group (patients with diabetes diagnosis) and was not available for the overall patient population. This prevented the assessment of this variable, which is an accurate predictor of glycemic control status, as a risk factor for PJI development.

There are also some strengths related to this study that need to be mentioned. To our knowledge, this is the second study evaluating risk factors for PJI, specifically after RSA surgery and controlling for potential confounders using a multivariate analysis. Therefore, the present study aims to fill an important gap in the available literature. Secondly, the present study has a considerably longer follow-up duration compared to the mentioned study [24]; thus, mid- to long-term results have been reported. The same standard surgical technique was performed by a single surgeon, and all patients were followed up by the same protocol, which can also be mentioned as another strength of the study.

## 5. Conclusions

PJI is one of the major concerns following RSA, which may cause devastating outcomes. However, current knowledge is markedly limited and mostly relies on evidence derived from studies about hip and knee arthroplasty. The present study fulfills this gap and guides shoulder surgeons for accurate preoperative risk assessment and patient management. Our findings showed that increased preoperative CRP levels and diabetes were associated with PJI following RSA surgery. Secondly, a history of diabetes was found to be an independent risk factor for PJI development, with an almost 5-fold increased risk. Further research specific to RSA, with a high evidence level, is still needed to comprehensively elicit risk factors related to PJI following RSA.

## Figures and Tables

**Table 1:** Diagnostic criteria described in 2018 by the International Consensus Meeting on Orthopedic Infections for periprosthetic joint infection (PJI).

Major Criteria (Definite PJI):	
Presence of a sinus tract to the prosthetic implant	
Presence of gross intra-articular pus	
Two positive tissue cultures for identical virulent organisms	
**Minor criteria**	**Points**
Unexpected wound drainage	4
Single positive tissue culture with virulent organism	3
Single positive tissue culture with low-virulence organism	1
Second positive tissue culture (identical low-virulence organism)	3
Humeral loosening	3
Positive frozen section (5 PMN in ≥5 high-power fields)	3
Positive preoperative aspirate culture (low or high virulence)	3
Elevated synovial neutrophil percentage (>80%) ^a^	2
Elevated ESR (>30 mm/h) ^a^	2
Elevated CRP level (>10 mg/L) ^a^	2
Elevated synovial α-defensin level	2
Cloudy aspiration fluid	2
**Minor criteria evaluation**	
≥6 points with the identified organism	Probable PJI
≥6 points without identified organism	Possible PJI
<6 points and	
Single positive culture with virulent organism	Possible PJI
2 positive cultures with low-virulence organism	Possible PJI
Negative culture or single positive culture with low-virulence organism	Unlikely PJI

Abbreviations: PJI: periprosthetic joint infection, PMN: polymorphonuclear leukocyte, ESR: erythrocyte sedimentation rate, CRP: C-reactive protein. ^a^: Available in 6th week after most recent surgery.

**Table 2:** Univariate analysis of demographic and clinical data.

Variable	Group I (n = 289)	Group II (n = 13)	*p* Value	Mean or Median Difference (95% CI)	Odds Ratio (95% CI)
Age (years), mean ± SD	70.0 ± 9.8	68.0 ± 15.8	0.585 ^a^	−2.0 ^e^ (−11.0 to 6.0)	N/A
Follow-up duration (months), mean ± SD	60.2 ± 24.7	52.3 ± 25.0	0.204 ^a^	−11.5 ^e^ (−22.6 to 4.43)	N/A
Gender, n (%) Male	65 (22.5)	5 (38.5)	0.188 ^b^	N/A	2.2 (0.68 to 6.8)
Female	224 (77.5)	8 (61.5)			
BMI (kg/m^2^), mean ± SD	29.0 ± 4.0	28.6 ± 2.6	0.852 ^a^	−0.6 ^e^ (−1.78 to 1.3)	N/A
Duration of hospital stay (days), mean ± SD	2.7 ± 1.1	3.2 ± 1.2	0.155 ^a^	0 ^e^ (0 to 1.0)	N/A
Duration of surgery (minutes), mean ± SD	128.2 ± 26.9	134.0 ± 28.9	0.489 ^c^	−5.82 ^f^ (−23.5 to 11.9)	N/A
Preoperative hemoglobin level (g/dL), mean ± SD	12.4 ± 1.7	12.0 ± 1.6	0.321 ^a^	−0.5 ^e^ (−1.4 to 0.3)	N/A
Preoperative hematocrit level (%), mean ± SD	38.1 ± 4.4	37.5 ± 5.6	0.690 ^c^	0.64 ^f^ (−2.77 to 4.06)	N/A
Preoperative CRP level (mg/L), mean ± SD	5.2 ± 13.9	10.3 ± 10.4	**0.001 ^a^**	3.0 ^e^ (1.7 to 9.4)	N/A
Preoperative ESR (mm/h), mean ± SD	16.8 ± 15.8	21.7 ± 20.5	0.545 ^a^	−3.0 ^e^ (−6.0 to 12.0)	N/A
Preoperative glucose level (mg/dL)	98.6 ± 18.9	100.9 ± 24.9	0.664 ^c^	−2.36 ^f^ (−13.0 to 8.31)	N/A
Diagnosis for RSA surgery, n (%)			0.963 ^d^		
CTA	132 (45.7)	5 (38.5)		N/A	N/A
PHF	71 (24.6)	4 (30.8)			
Glenohumeral OA	36 (12.5)	1 (7.7)			
Fracture sequelae	12 (4.2)	1 (7.7)			
AVN	17 (5.9)	1 (7.7)			
fARCR	21 (7.3)	1 (7.7)			
Intraoperative local teicoplanin powder, n (%)			0.389 ^b^	N/A	
no	174 (60.2)	6 (46.2)			1.77 (0.58 to 5.39)
yes	115 (39.8)	7 (53.8)			
Preoperative UTI, n (%)			0.703 ^b^		
no	239 (82.7)	12 (92.3)		N/A	0.39 (0.05 to 3.13)
yes	50 (17.3)	1 (7.7)			
Tobacca use, n (%)			0.546 ^d^		
no	250 (86.5)	12 (92.3)		N/A	0.53 (0.07 to 4.2)
yes	39 (13.5)	1 (7.7)			
COPD history, n (%)			0.247 ^d^		
no	282 (97.6)	12 (92.3)		N/A	3.36 (0.38 to 29.5)
yes	7 (2.4)	1 (7.7)			
Diabetes history, n (%)			**0.007 ^b^**		
no	217 (75.1)	5 (38.5)		N/A	4.8 (1.5 to 15.2)
yes	72 (24.9)	8 (61.5)			
Hypertension, n (%)			0.259 ^b^		
no	162 (56.1)	5 (38.5)		N/A	2.04 (0.65 to 6.39)
yes	237 (43.9)	8 (61.5)			
Coronary artery disease, n (%)			0.066 ^b^		
no	255 (88.2)	9 (69.2)		N/A	3.33 (0.97 to 11.4)
yes	34 (11.8)	4 (30.8)			

Abbreviations: BMI: body mass index, CRP: C-reactive protein, ESR: erythrocyte sedimentation rate, RSA: reverse shoulder arthroplasty, CTA: cuff tear arthropathy, PHF: proximal humeral fracture, OA: osteoarthritis, AVN: avascular necrosis, fARCR: failed previous arthroscopic rotator cuff repair, UTI: urinary tract infection, COPD: chronic obstructive pulmonary disease, CI: confidence interval, N/A: not applicable. ^a^: Mann-Whitney U test, ^b^: Fisher’s exact test, ^c^: Unpaired samples *t*-test, ^d^: Chi-square test, ^e^: median, ^f^: mean. Bolded *p* values indicate statistical significance.

**Table 3:** Multivariate logistic regression analysis to detect independent risk factors for periprosthetic joint infection.

Variable	Odds Ratio	95%CI	*p* Value
Preoperative CRP level	1.016	0.98 to 1.04	0.219
Diabetes history	4.852	1.56 to 16.6	**0.007**

Abbreviations: CRP: C-reactive protein, CI: confidence interval. Bolded *p* values indicate statistical significance.

## Data Availability

The raw data supporting the conclusions of this article will be made available by the authors upon request.

## Abbreviations

The following abbreviations are used in this manuscript:

RSA	Reverse shoulder arthroplasty
PJI	Periprosthetic joint infection
CRP	C-reactive protein
CTA	Cuff tear arthropathy
OA	Osteoarthritis
AVN	Avascular necrosis
PHF	Proximal humeral fracture
fARCR	Failed arthroscopic rotator cuff repair
ROM	Range of motion
UTI	Urinary tract infection
IV	Intravenous
ICM	International Consensus Meeting
ESR	Erythrocyte sedimentation rate
HbA1c	Hemoglobin A1c
EBJIS	European Bone and Joint Infection Society
IL-6	Interleukin-6
WBC	White blood cell
TNF-α	Tumor necrosis factor-α
NLR	Neutrophil/lymphocyte ratio
SII	Systemic inflammation index
COPD	Chronic obstructive pulmonary disease
